# The London Regional Major Trauma System: A Literature Review of Epidemiology and Current and Future Challenges

**DOI:** 10.7759/cureus.73998

**Published:** 2024-11-19

**Authors:** Sanjeevi Bharadwaj, Subasri Balasubramanium

**Affiliations:** 1 Trauma and Orthopedics, The Royal London Hospital, Barts Health NHS Trust, London, GBR; 2 Trauma Sciences, Blizard Institute, Queen Mary University, London, GBR; 3 Emergency Medicine, West Midlands Deanery, Birmingham, GBR

**Keywords:** covid, helicopter emergency medical service, hems, inclusive trauma network system, london trauma network system, major trauma center, tarn, trauma, trauma unit, triage

## Abstract

Trauma has been one of the world’s most common causes of death among younger age groups. In the UK, a lack of an organized and streamlined approach was reported in the management of traumatic injuries and patients involved in trauma cases in the UK. Therefore, a major trauma network system was devised to address these issues in line with other trauma systems around the world. This was followed by the establishment of the London trauma network with four major trauma centers (MTCs) spread across the London region, with each of these MTCs connected to a group of smaller hospitals called trauma units (TUs). The functioning of all these MTCs is governed by national standards. A trauma system involves an inclusive network of trauma care providers. This network includes pre-hospital care services, hospitals receiving acute trauma admissions, and post-hospital rehabilitation services working in close coordination with social care services and community services to achieve optimal use of these resources to deliver the correct treatment at the correct time by specialist MTCs. Specifically designed major trauma triage tools are used by the prehospital teams to identify severely injured patients, and those who have been identified as triage-positive patients are airlifted and transferred by air ambulances to the MTCs within 60 minutes. For instance, the Royal London Hospital’s Helicopter Emergency Medical Service (HEMS) is the pride of London’s trauma network system and plays an important role in reducing the transfer time. First, MTCs are expected to handle massive volumes of trauma, and they are constantly under pressure to meet national standards. Therefore, it becomes necessary for the smaller TU hospitals to ensure the patients who are to be transferred have adequately undergone triage through established specific local triage protocols, which could, however, result in under-triage errors because of misjudgment of major injuries as minor injuries, which subsequently get less priority while managing minor trauma. Second, the changing landscape of surgical training with increasing demands and pressure on the system has put the trainees in a dilemma about the sustainability of the current training curriculum. Emphasis should be given to simulation-based training, which could address the issues of reduced theater time and could work as a good alternative, both for learning and teaching. Third, the lack of self-directed therapy that is tailored to meet a patient’s demands has resulted in a disorganized delivery of rehabilitation services for patients suffering from polytrauma. However, like any system trying to make a paradigm shift, having pros and certain distinctive characteristics, the trauma system also has certain deficits, which, when appropriately addressed, as mentioned earlier, may ensure smoother functioning of the trauma system.

## Introduction and background

Trauma has been one of the world’s most common causes of death among younger age groups [1]. It was recognized in many reports that such deaths were a result of a lack of an organized approach to dealing with trauma injuries and patients involved in road traffic accidents in the UK. Therefore, these reports recommended the establishment of major trauma centers (MTCs) with trauma networks along with improved training of paramedics with improved communication and more effective prehospital care [2]. The NCEPOD 2007 report “Trauma, who cares?" reported serious errors and shortcomings in delivering structured trauma care in the UK [3]. The report also suggested that the standard of care received by more than half of the patients with trauma injuries was less than the minimum standards, with a recurrence of avoidable deaths [3]. Before the advent of the trauma systems in the United Kingdom (UK), patients involved in trauma injuries were previously transported to the closest hospital, irrespective of the intensity and extent of the trauma, which resulted in the loss of precious time, which should have been utilized effectively by having a well-structured approach to this problem [1,2]. By the latter half of the 20th century, it became clear that to reduce the number of deaths due to major trauma, there was a need to have centralized institutes with resources and health experts organized in a way that could improve the outcome of trauma care [1]. The UK’s trauma system was developed to address these issues and was to be in line with various other trauma systems around the world.

## Review

Epidemiology of trauma in the UK and London

Trauma has been one of the world’s most common causes of death among the younger age groups. Over the last 10-15 years in England, there has been a significant increase in the incidence of recorded trauma cases, with a concurrent annual increase in the incidence of trauma-based hospital admissions among ages 10-24 years between 2010 and 2016 [4]. Over the last decade, it has been observed that suicide and trauma have become the leading causes of death among males between the ages of 35 and 49, unlike in the initial years of the millennium, when the number of deaths per year in males was thrice the number of those in females. Furthermore, it has also been noted that in 2018 alone, 27.1% of deaths among males 20 to 34 years of age were due to suicide and injury, as opposed to 16.7% among females of the same age group.

An estimate by the National Audit Office suggested that every year, there are more than 20,000 cases of major trauma in the UK, leading to more than 5,400 cases of death and permanent disability requiring long-term care. Another 2800 cases follow similar lines of management, although they do not meet the precise definition of major trauma. Studies have also shown that the most common cause of admissions from trauma in London is due to blunt injuries resulting from falls from heights and Road Traffic Accidents (RTA’s) in particular, followed by penetrating injuries at 79% and 21%, respectively [5].

Meanwhile, research conducted on the epidemiology of adolescent trauma in England based on TARN Data from 2008 to 2017 reported that in the UK, the leading cause of trauma among the younger groups of 10-15 years of age is road traffic accidents, which had accounted for 50.3% of all cases with a male predominance, followed by falls from heights and penetrating injuries. On the contrary, among older age groups between the ages of 16 and 24 years, the most often cause of trauma was self-inflicted intentional injuries [4].

Trauma system and its benefits

A trauma system involves an inclusive network of trauma care providers. This network includes pre-hospital care services, hospitals receiving acute trauma admissions, and post-hospital rehabilitation services working in close coordination with the social care services and the community so that there is an optimal use of these resources to deliver appropriate treatment at the correct time by specialist MTCs. Around the world, various trauma systems have evolved over the years, with recorded evidence of improved functional outcomes with the management of trauma cases while also decreasing the mortality rate involved with traumatic injuries [1]. To improve the effective delivery of the provision of major trauma care, an inclusive trauma network system was implemented in London, and three MTCs were established in April 2010. A fourth center followed this in April 2011 in London [1,2,6]. Following London’s lead, a network of hospitals has been developed based on geography, available facilities, and transfer times, leading to the formation of a closed-loop network involving MTCs, trauma units (TUs), and local emergency hospitals, district general hospitals, or acute hospitals [7]. Specifically designed major trauma triage tools are used by prehospital teams to identify severely injured patients, and those who have been identified as triage-positive patients are airlifted and transferred by air ambulances to the MTCs within 60 minutes. The Royal London Hospital’s Helicopter Emergency Medical Service (HEMS) is the pride of London’s trauma network system and plays an important role in reducing transfer time. The UK boasts of a trauma system with 27 MTCs, out of which 11 are for adults and children, 11 for adults and five for children. Of these, the London Trauma Network includes four trauma networks (North West London, North East London and Essex, South West London and Surrey, South East London and Kent trauma networks) [6,7]. The functioning of these MTCs is governed by national standards, which include round-the-clock availability of a specialized trauma team led by consultants to ensure that a computed tomography (CT) scan is performed within 60 minutes of the arrival of high-priority patients with a Glasgow Coma Scale 8 or below. These standards also dictate that these severely injured patients receive tranexamic acid within the first three hours after the injury, along with massive transfusion protocols in place, ready to be activated at a moment's notice to manage hemodynamically unstable patients. There are also standards regarding the prescription of the rehabilitation process on discharge. To ensure that the MTCs and their respective trauma networks adhere to these national standards, they are supposed to conduct regular audits and submit their data to the Trauma Audit Research Network (TARN) [5].

Benefits of the London Regional Major Trauma System

Shreds of evidence show that there was a 23% reduction in the mortality of patients involved in major trauma following the establishment of the inclusive London regional trauma network system, thereby making a significant difference in the overall management and outcome of major trauma injuries [6,8]. Patient transfer to the MTCs following a major trauma has become more streamlined and safer with the introduction of specific protocols. These protocols have been designed based on the mechanism of injury, assessment of patient physiology, individual patient factors, and anatomy of apparent injuries to assist in decision-making while transferring major trauma patients to an MTC [6,9]. The close collaboration and coordinated approach among the emergency services, MTCs, TUs, and community service providers helps deliver high standards of coordinated and collaborative care to the patients involved in major trauma incidents across the spectrum of the trauma pathway concerning initial injury and rehabilitation [6]. The overall patient outcome and quality of care delivered at the MTCs have significantly improved and increased early survival rates with a coordinated and collaborative approach to the delivery of specialist multi-disciplinary care, involving prompt initial management of major trauma patients along with access to radiological modalities and operating theaters [6,10]. To ensure high standards of care and outcomes for patients involved in major trauma, MTCs equipped with the necessary expertise, resources, and dedicated trauma teams with continuous access to consultant-led trauma service with multi-specialty care (such as general, emergency medicine, vascular, trauma, and orthopedics, plastic, spinal, maxillofacial, cardiothoracic, and neurological surgery, critical care, and interventional radiology) [6]. Literature shows that a multidisciplinary consultant-led approach to the management of trauma patients reduces injury to intervention time, with rapid access to evaluation, resuscitation, investigations, and interventions [6,11]. A multicentric study involving 207 patients by Driscoll and Vincent showed that the presence of a well-coordinated and dedicated major trauma team reduced the resuscitation time to 56 minutes from 122 minutes [6,12]. With an estimated 45% of CT scans performed within 30 minutes of arrival, following the introduction of the London major trauma system, early round-the-clock access to resident consultant-led imaging services and guidance with trauma CT has played an important role in the early assessment, rapid diagnosis, effective triage, and management of complex polytrauma patients with life-threatening injuries [2,6,13]. The round-the-clock availability of a well-integrated imaging service amongst the MTCs and TUs within the regional trauma network through the Image Exchange Portal (IEP) system allows more efficient and quicker, time- and cost-effective delivery of a specialist radiological opinion or advice [6].

Challenges for the London Regional Major Trauma System

Notwithstanding the fact that there has been significant cohesiveness and improvement over the years in the management of major trauma patients since the implementation of the London major trauma network system, it has been facing many challenges. The COVID-19 pandemic has posed a major challenge to the trauma network services in the delivery of trauma services across the London region, resulting in the reconfiguration of the trauma services to facilitate the smooth delivery of COVID-19 medical care at the expense of an already overstretched trauma network system, which was already under considerable pressure [6,14]. During the COVID-19 pandemic, the major trauma network system experienced significant pressure due to infrastructural demand, overstretching of infection control resources, and understaffing either due to re-deployment or illness, thereby necessitating the major trauma network systems to reshape their guidelines and develop rapidly adaptive strategies to suit the available resources and local infrastructure of the trauma network [6,15]. However, within no time, trauma and elective surgical provisions came under immense pressure with the postponement of elective procedures and reconsideration of the threshold for trauma surgeries due to operation theaters being altered into an extension of critical care units and redeployment of anesthetic staff [6,14]. Although some fractures were initially managed conservatively, they resulted in requiring an operative intervention later due to potential displacement, resulting in non-unions or malunions with delayed presentations due to the COVID-19 pandemic [6,14]. A major part of the challenge was also faced by the surgical trainees in the UK across all major trauma networks. There was a possible disparity in the operative experience gained by the trainees during the COVID-19 pandemic, with the trainees at the MTCs performing 10% more trauma surgeries than those in the TUs [6,16]. Although the exact re-distribution of trauma patients across the London network is unclear, Hipps et al. (2015) reported a 19% increase in indexed trauma procedures like intramedullary nailing in two of the MTCs in the Northern trauma network as opposed to a 16% reduction in eight of the TUs [6,16].

Recommendations to address the challenges in the current trauma provision in the London Regional Major Trauma Network

Initially, the guidelines suggest that all MTCs in England should be capable of handling around 400 cases of major trauma on an annual basis. This would at least enable and equip the MTCs to cater to the needs of a population of around three million [1]. This results in MTCs like the Royal London Hospital coming under the immense pressure of managing a high volume of trauma while constantly striving to deliver world-class outcomes to meet the dictated national standards. This is because MTCs like the Royal London Hospital are, by definition, larger advanced training and teaching institutes providing injury prevention initiatives, education, training, and research [1].

It, therefore, should be a priority for smaller TUs in the London Regional Trauma Network to have specific triage protocols in effect to manage and handle minor trauma patients with an injury severity score of <15, as there is a high possibility of these minor injuries getting a much lower priority when compared to patients with major and more complex trauma injuries [9]. This would, in turn, affect the outcome for the transferred patient, who would rather have been managed much more effectively at the TU itself [1]. On the other hand, there may be incidences of undertriage errors that may result in severely injured patients being taken to a TU, in which case these smaller centers can step up and perform urgent life-saving interventions before transferring them to the MTC with immediate transfer protocols between the TU and MTC [1].

Second, the changing landscape of surgical training has been increasing the pressure on the system, which had already been there even before the COVID-19 pandemic and had increased following the pandemic [7]. This has put the trainees at the Royal London Rotation in a dilemma as to whether the current training curriculum could be sustainable with increasing demands and pressure on the system. The reduction in surgical exposure in theater time has inadvertently impacted the trainees, and this situation could worsen in the future if another major unforeseen pandemic occurs. Therefore, there is an urgent need to review the current surgical curriculum, which is heavily based on traditional methods [6]. In an attempt to address the dilemma that is posing the UK’s surgical trainees and trainers concerning the sustainability and adequacy of the current surgical training due to reduced theater time, which would harm the smooth surgical training in the wake of major future pandemics [6]. There is a serious need to modernize the current surgical curriculum with simulation-based training [6,17]. There is already an existing format in the Royal Colleges for basic surgical skills, the Advanced Trauma and Life Support Training (ATLS) and Care of Critically Ill Surgical Patients (CCriSP), which are simulation-based training courses. Including simulation-based learning and training methods using virtual reality for training in surgical procedures could offer a safe yet realistic alternative for both learning and teaching in the UK’s highly competency-based surgical training pathway [6,17].

Third, there has been an existential deficiency in the delivery of organized rehabilitation with specialist-led services, especially for severely injured patients and those with high demands following a complex major trauma surgery. This could be attributed to the lack of a centralized rehabilitation pathway aimed at more self-directed therapy that is tailored to meet a patient’s demands. This is achieved through synchronized redistribution of all the available resources to bridge the gap between the high-demand patients and the rehabilitation specialists, thereby improving engagement from both sides [1,9,18].

With such a framework in place, the rehabilitation process is delivered in two phases. The acute phase begins in the hospital during the immediate postoperative period, followed by the continuation phase, which continues at the local hospital even after the patient's repatriation. On the other hand, if the patient chooses when it is safe to be discharged to his home, the rehabilitation can be continued through community services as well [9].

To ensure a smooth transfer of care following a patient’s completion of treatment in the MTC, rehabilitation services must be integrated at local specialized facilities. This can improve rehabilitation outcomes provided there is early intensive involvement of all the stakeholders involved in trauma care [9].

## Conclusions

Trauma incidents rank among the leading causes of death in younger populations. This report indicated that most patients experiencing major trauma did not receive care that met the necessary standards. To enhance major trauma care delivery, London implemented a comprehensive trauma network system, launching three MTCs in April 2010. A fourth center followed in April 2011 within the city. The operations of all these MTCs were regulated by national standards. However, like any system undergoing significant changes, the trauma system faces certain deficits or challenges; addressing these appropriately may facilitate its effective functioning. Ultimately, to maintain high care standards, trauma systems must be continuously monitored through regular audits and quality improvement initiatives, with findings submitted to the trauma registry for evaluation by the TARN.
